# Short Communication: Opportunities and Challenges for Early Person-Centered Care for Older Patients in Emergency Settings

**DOI:** 10.3390/ijerph182312526

**Published:** 2021-11-28

**Authors:** Andrea N. Jensen, Ove Andersen, Hejdi Gamst-Jensen, Maria Kristiansen

**Affiliations:** 1 Department of Clinical Research, Copenhagen University Hospital—Amager and Hvidovre, Kettegaard Allé 30, 2650 Hvidovre, Denmark; ove.andersen@regionh.dk (O.A.); Hejdi.gamst-jensen@regionh.dk (H.G.-J.); 2 Department of Public Health, Faculty of Health and Medical Sciences, University of Copenhagen, Oester Farimagsgade 5, 1014 Copenhagen, Denmark; makk@sund.ku.dk; 3 Department of Emergency Medicine, Copenhagen University Hospital—Amager and Hvidovre, Kettegaard Allé 30, 2650 Hvidovre, Denmark; 4 Department of Clinical Medicine, University of Copenhagen, Blegdamsvej 3B, 2200 Copenhagen, Denmark; 5 Center for Healthy Aging, Faculty of Health and Medical Sciences, University of Copenhagen, Blegdamsvej 3B, 2200 Copenhagen, Denmark

**Keywords:** person-centered care, emergency department, patient engagement, frail older adults, qualitative research, communication

## Abstract

The provision of person-centered care (PCC) for older adults in emergency settings is important. This short communication explores the complexity of providing comprehensive PCC for older adults in emergency settings, based on a synthesis of existing literature and empirical data from a small-scale case study on the potential of improving patient engagement in a Danish emergency department (ED). Our findings highlight overall positive attitudes towards PCC, as patient engagement is perceived as important and feasible during the waiting hours that older patients experience. However, the key challenges include barriers in organizational structures and cross-sectoral care coordination. We conclude that staff education, optimized care coordination across sectors, and increased involvement of geriatric nurses may enhance the provision of PCC for older, complex adults in EDs. We further conclude that future research into the feasibility and effects of structured approaches for providing PCC in EDs is needed, including exploration of organizational models for PCC.

## 1. Introduction

Population aging has implications for the overall provision of health care, including in the settings of emergency departments (EDs), requiring adjustment to the needs of an increasing number of older patients [1,2,3,4]. Multimorbidity, polypharmacy and psychosocial concerns result in more complex care needs among older ED patients [5,6,7]. Identifying and addressing these complex care needs is key to prevent unnecessary disease progression, decrease in quality of life, and unwarranted health care utilization [8]. Studies show that, following ED visits, older patients have an increased likelihood of revisits [9], often experience a decline in their functional ability [10], and have higher mortality [3,10], indicating room for improvement in care delivery. To ensure optimal care and safe discharge in this population, person-centered care (PCC), defined as care that is respectful of and responsive to individual patient preferences, needs, and values, is needed [11,12,13]. Thus, we follow the definition of PCC that reflects the 2001 statement from the Institute of Medicine, which states the following: “Health care should be patient-centered—providing care that is respectful of and responsive to individual patient preferences, needs, and values and ensuring that patient values guide all clinical decisions.” [14]. PCC approaches engage with patients in a holistic manner, considering all life aspects of the patient [15], and, further, take the contextual circumstances under which the care is provided into consideration [13].

Centering health care provision around individual needs promotes flexibility in health care and leads to improved patient satisfaction [12]. Although PCC, overall, and shared decision making (SDM), more concretely, have received increasing attention in EDs, research within this field has mostly focused on end-of-life conversations [16], high-risk older patients [17], specific diseases or symptoms [18,19], and populations other than older patients [20,21,22].

This short communication aims to elucidate the complexity of providing PCC for older patients in EDs, and intends to discuss the opportunities and challenges on this matter. The discussion is based on a synthesis of existing literature within the field, and empirical inputs from a small-scale case study, conducted at an ED in the Capital Region of Copenhagen, Denmark.

## 2. Materials and Methods

### 2.1. Setting and Approach

Data collection took place at an ED from September to January 2019, among nurses and a geriatric team situated in the ED, in the Capital Region of Copenhagen, Denmark. The ED has focused on ensuring PCC for particularly vulnerable older adults with complex care needs. As a part of the approach to care provision, a small-scale case study was conducted to explore opportunities for more structured PCC conversations in EDs, inspired by older adults’ own assessments of their plans and wishes [23].

### 2.2. Data Collection and Analysis

Empirical data consisted of participant observation, informal interviews with nurses and physicians, and interviews with ED nurses and geriatric nurses.

Participant observation was carried out with the main author accompanying different nurses (n = 14) to gain in-depth contextual insights into circumstances shaping clinical encounters, and to qualify the interview guide used in the proceeding interviews [24]. Approximately 60 encounters were observed. An observation guide focusing on organizational structures, practices, workflow and PCC was used to ensure systematic observations [25]. Field notes were taken during observations and were expanded upon after each fieldwork.

Further, two individual semi-structured interviews with ED nurses, and two focus group interviews with four ED nurses and three geriatric nurses, respectively, were carried out in facilities near the ED. In total, eight nurses were interviewed. The aim was to elucidate perspectives on patient engagement and PCC in the ED. Permission to conduct the fieldwork was obtained from the nurse manager before the data collection and an information letter was sent to the ED inviting nurses to participate. The study was presented at the ED as a part of the recruitment process. Informal interviews with 20 ED nurses and physicians during the fieldwork illuminated the complexity of providing PCC in EDs.

Interviews were audio-recorded and transcribed verbatim. Observational data and preliminary findings were discussed in the author group to ensure intercoder reliability [26,27]. Following discussions, the empirical data, including data from the informal interviews, were analyzed using thematic network analysis inspired by Attride-Stirling [28].

### 2.3. Ethical Considerations

Before each interview, nurses were informed about the study objective and their right to withdraw at any time. All participants gave informed consent. The Institutional Review Board of The Danish Data Protection Agency (journal number P-2019-823) approved this study.

## 3. Results

**Figure 1:**
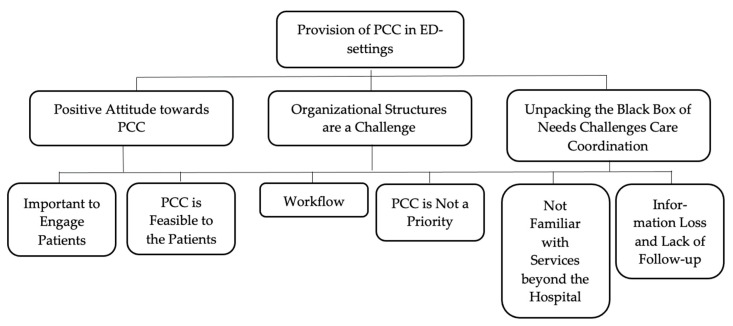
Thematic network: illustration of the thematic network that emerged from the analysis of the empirical data (observations, interviews and informal interviews).

Overall, the following three themes emerged from the analysis of the empirical data: (1) positive attitude towards PCC; (2) organizational structures are a challenge; (3) unpacking the black box of needs challenges care coordination. Each theme contained several subthemes. Figure 1 illustrates the thematic network that emerged, and Table 1 provide an overview of the themes including excerpts from the empirical data.

### 3.1. Positive Attitude towards PCC

Overall, the nurses had a positive attitude towards PCC as an approach for ED care and emphasized the importance of engaging actively with older patients. The nurses agreed that PCC would enable a deeper understanding of the needs of the individual patients, as unhurried PCC conversations would allow for questions concerning areas other than those usually discussed. Further, some nurses believed that it would be feasible for older patients to engage in unhurried conversations and needs assessments, potentially preventing the likelihood of revisits. Moreover, our field notes revealed that the majority of older patients who presented in the ED had both the physical and mental ability to be engaged.

### 3.2. Organizational Structures Are a Challenge

Despite the positive potential identified by nurses, several challenges appeared. In particular, the setting and workflow of the ED were characterized by time constraints, rapid assessments of patient needs, and the need for prioritizing between tasks and patients. We found that nurses were reluctant to engage in more comprehensive conversations and would rather focus on acute treatment. The nurses highlighted that, although they perceived PCC as favorable and feasible for older patients, they would not be able to prioritize these more structured and unhurried conversations due to their rapid workflow.

### 3.3. Unpacking the Black Box of Needs Challenges Care Coordination

Furthermore, concerns related to unpacking the ‘black box’ of needs among older patients were raised. The nurses expressed that their job relates to uncovering and addressing the medical needs of patients, and that they are not familiar with the services offered beyond the hospital setting. This challenges care coordination across sectors, and was perceived to be a key challenge that prevented the uptake of PCC conversations. Loss of information and lack of a follow-up were particular concerns.

## 4. Discussion

There is a need to approach PCC in EDs more systematically and comprehensively, to unlock the likely positive effect on care and more appropriate resource allocation for the growing population of older patients in EDs [5,11,29,30].

### 4.1. Engagement of Patients and Long Hours of Waiting as Opportunities

In this study, nurses acknowledged the importance of PCC and emphasized the potentials of the approach. This is in line with previous research exploring how to facilitate the transition to PCC in clinical practice [31]. PCC provides several opportunities, including gaining insights into patients’ individual lives and circumstances. Additionally, more structured PCC conversations may facilitate the systematic engagement of patients in a more comprehensive manner than is often the case in EDs [30,32]. As a result, it would be recognized that life domains that extend beyond traditional disease-management dimensions are of importance for care provision for older patients [13,23]. This may help to avoid interpersonal differences when engaging in PCC conversations with older patients. Although contextual circumstances, such as long waiting hours for patients, constitute a challenge for efficiency and patient satisfaction in clinical encounters, they could also be used as opportunities to enable older patients to be engaged in conversations revolving around their values and goals, to inform PCC approaches and improve care trajectories [30,33].

### 4.2. Organizational Structures, Such as Workflow and Lack of Prioritizing PCC, as Challenges

The ED environment, with a high-intensity workflow, is characterized by a constant uncertainty of what type of patient will arrive next, and a need for rapid prioritization between tasks and patients, which challenges unhurried PCC conversations [4,34]. These conditions may make health care professionals reluctant to engage in PCC conversations about individual life circumstances with older patients, with reference to the potential interruptions caused by another acutely ill patient. This is further reflected in the prioritization of acute treatment among ED nurses. This finding is supported by Kirk et al. [34], who argue that ED nurses consider themselves to be experts in acute treatment and in maintaining patient flow. The hierarchy of tasks within the ED setting, with priority given to the provision of highly specialized treatment, affects the possibility of engaging with the complex care needs that appear in more comprehensive patient–provider conversations, which is a finding that is also reflected elsewhere [31]. However, PCC approaches acknowledge that the biography and life circumstances of the patient shape the experiences and, ultimately, outcomes of the health care provided. Following this, McCormack (2003) argues that PCC for older adults must encompass more than practical expertise, thereby ensuring that individuals are supported and empowered to be engaged in defining and altering their own health care trajectory [13].

### 4.3. Information Loss and Uncertainties with Services beyond the Hospital Setting Challenges Care Coordination

A third key challenge relates to concerns surrounding the unpacking of the ‘black box’ of older patients’ needs, and coordination of the cross-sectoral transition of care, which is hampered by different organizational structures. In Denmark, municipalities and general practitioners provide a variety of services that may correspond with the needs, values, and preferences of older patients in the ED. This study highlights the complexity of providing PCC for older adults in ED settings, especially regarding cross-sectoral collaboration. Health care professionals may not be familiar with the existing services beyond the hospital setting, which are needed for accommodating for the totality of often complex health and social needs, raising insecurities about whether the non-medical needs of older adults can be accommodated. In addition, time constraints in the ED challenge care coordination. This barrier has also been noted by Lennox et al. (2018), who argue that a lack of adequate care coordination is exacerbated in older populations due to their complex care needs [6]. Although insufficient cross-sectoral communication may lead to an uncoordinated and inadequate transition of care and follow-up, EDs hold a unique position, as they bridge multiple settings across sectors, such as prehospital, home, inpatient, and outpatient [35]. This position allows the ED to be involved in facilitating PCC across sectors, which is important as various combinations of chronic diseases and social determinants may affect achievement of the goal. Therefore, transforming care to fit the individual older patient requires interdisciplinary input and cross-sectoral collaboration [15,33]. Effective cross-sectoral coordination and communication are especially important when designing individualized discharge plans, as the plans may ensure a safe discharge, reduce the length of hospital stays, and reduce readmissions among older medical patients [36]. Hence, more unhurried PCC conversations may unpack the ‘black box’ of older patients’ needs, and resources that provide better and more PCC consequently feed into targeted and responsive follow-up care [11,29].

### 4.4. Future Directions

The insights reflected in this short communication illustrate the complexities, but also the potential, of providing PCC for older patients in emergency settings, albeit with key organizational challenges to be overcome. However, this short communication also points towards future directions and raises questions that future research within the field should address:

- Future studies into the feasibility and effects of structured approaches for providing PCC for older adults in ED settings are necessary.
- Future research into the identification of organizational models for PCC is necessary, including an exploration of how and when, during the ED visit, PCC is achievable and which health care professionals should be involved.
- Providing PCC for older adults in EDs requires staff education, geriatric-specific knowledge, and optimized coordination across sectors.
- Increased involvement of the geriatric nurses situated in the ED may facilitate PCC to a greater extent, both within the ED and across sectors.

## 5. Conclusions

It is important to assess and engage with older patient’s needs, values, and preferences as part of emergency care for this growing and complex population group. However, providing PCC in EDs is challenging due to the high patient flow, unpredictable tasks, and limited time available for unpacking the ‘black box’ of older patients’ needs. Further, follow-up requires cross-sectoral collaboration, which is found to be challenging. Staff education, optimized care coordination across sectors, and increased involvement of the geriatric nurses may enhance the provision of PCC for older, complex adults in EDs. Future research into the feasibility and effects of structured approaches for providing PCC in ED settings is needed, including the exploration of organizational models for PCC.

## Figures and Tables

**Table 1:** Overview of themes.

	Examples	
Emerging Theme	Excerpts from Interviews and Informal Interviews	Clinical Example from Fieldwork
**Positive Attitude towards PCC** - Important to Engage Patients and PCC is Feasible to the Patients	“(…) I find it really important to try to engage the patients to uncover how we may accommodate their needs in the best possible way according to the resources currently available.” (Nurse 2). “[Using a PCC approach] We would get to know the patients in another way than usual. Our regular questions differ from nurse to nurse: it all depends on what you ask and what you respond to. (…) So, it could be beneficial if we became more aware of the issues and payed more attention to the older adults.” (Nurse 6). “So, I believe that it [PCC conversations] is feasible for the patients. If some of the things that are the most important to them can be fulfilled—then I think it can create better care and maybe prevent some things, such as hospitalization. Maybe they [the patients] can better cater to some of the things that they are encouraged to do during hospitalization following a PCC conversation (…)”. (Nurse 2).	Multiple older ED patients are staying in their respective beds for longer periods, enabling unhurried conversations without interruption from either nurses, physicians, or other health care professionals (Field notes, ED, November 2019). Yet again my experience suggests that PCC conversations would be relevant for several older ED patients who often have the physical and mental ability to engage and participate in a holistic conversation (Field notes, ED, November 2019).
**Organizational Structures are a Challenge** - Workflow and PCC is Not a Priority	“We do not only have one patient—we also have six others and different emergency rooms we must run to. And we don’t know what we’ll receive in five minutes. We have no scheduled tasks. (…) For us, it’s about prioritizing. I can easily postpone a blood glucose test if another patient needs my help more. I can easily postpone giving medicine until noon if some of my colleagues need help with something. So, it [PCC conversations] is not going to be prioritized—that’s what I’m trying to say.” (Nurse 4).	Entering the ED, I am met by a busy hallway with nurses walking back and forth. Pharmacists are walking from patient to patient with medication. Physicians are visiting patients and entering the staff office, where they confer with colleagues and write up medical records (Field notes, ED, September 2019). Today, the temporality is high—the nurses have several additional tasks beyond acute treatment, and less time to write up medical records. Little time is spent with the patients (Field notes, ED, November 2019). Person-centered care is not a priority. Acute treatment, on the other hand, is a priority. The nurse that I am accompanying today tells me that she asks herself: “Will this task save lives?”—her priorities are the medical tasks that can ultimately save lives (Field notes, ED, December 2019).
**Unpacking the Black Box of Needs Challenges Care Coordination** - Not Familiar with Services beyond the Hospital and Information Loss and Lack of Follow-up	“Someone has to follow up on the needs that we uncover [during PCC conversations]. And in the emergency department we do not know exactly what the municipality can offer and what can be accommodated (…).” (Nurse 3). “The municipality must accommodate it [the needs uncovered]. (…). And there is a communicative path between us and them [the municipality] (…) that’s a lot of work, and there are also a lot of things that can go wrong. And if we uncover unmet needs and say: ‘Well, we will pass this on to the municipality, if it’s okay with you?’, and then we pass it on, and the municipality cannot accommodate it, then there might be some disappointment associated with it.” (Nurse 2).	The geriatric nurses express concerns related to time spent when entering PCC conversations and further doubts as to whether the conversations with the older patients will reveal needs that they cannot accommodate. They express concern that they are not familiar with the existing services of the municipality, which complicates referral to cross-sectoral services (Field notes, geriatric team, September 2019).

## Data Availability

The data generated and/or analyzed during the current study are not publicly available due to the sensitive nature of the data.
